# Human cortical neural correlates of visual fatigue during binocular depth perception: An fNIRS study

**DOI:** 10.1371/journal.pone.0172426

**Published:** 2017-02-16

**Authors:** Tingting Cai, Huilin Zhu, Jie Xu, Shijing Wu, Xinge Li, Sailing He

**Affiliations:** 1 Centre for Optical and Electromagnetic Research, South China Academy of Advanced Optoelectronics, South China Normal University (SCNU), Guangzhou, China; 2 School of Information and Optoelectronic Science and Engineering, South China Normal University (SCNU), Guangzhou, China; 3 South China Normal University (SCNU), Guangzhou, China; 4 Department of Electromagnetic Engineering, Royal Institute of Technology, Stockholm, Sweden; The University of Melbourne, AUSTRALIA

## Abstract

Functional near-infrared spectroscopy (fNIRS) was adopted to investigate the cortical neural correlates of visual fatigue during binocular depth perception for different disparities (from 0.1° to 1.5°). By using a slow event-related paradigm, the oxyhaemoglobin (HbO) responses to fused binocular stimuli presented by the random-dot stereogram (RDS) were recorded over the whole visual dorsal area. To extract from an HbO curve the characteristics that are correlated with subjective experiences of stereopsis and visual fatigue, we proposed a novel method to fit the time-course HbO curve with various response functions which could reflect various processes of binocular depth perception. Our results indicate that the parietal-occipital cortices are spatially correlated with binocular depth perception and that the process of depth perception includes two steps, associated with generating and sustaining stereovision. Visual fatigue is caused mainly by generating stereovision, while the amplitude of the haemodynamic response corresponding to sustaining stereovision is correlated with stereopsis. Combining statistical parameter analysis and the fitted time-course analysis, fNIRS could be a promising method to study visual fatigue and possibly other multi-process neural bases.

## Introduction

Functional near-infrared spectroscopy (fNIRS) [1, 2] is a non-invasive technique that uses either continuous or frequency-modulated near infrared light to record activity-induced haemodynamic changes that reflect total haemoglobin (HbT), oxyhaemoglobin (HbO), and deoxyhaemoglobin (Hb) within the cerebral cortex. With good temporal resolution and reasonable spatial resolution, this technique has been widely adopted to record brain activation in response to cognitive or perceptual processes such as stereopsis [3, 4]. Stereopsis is the perception of depth by the brain in which both binocular and monocular cues are utilized. When viewing an object, the separation of the two eyes results in two retinal images, projected onto slightly different parts of the retina, known as retinal or binocular disparity. The fusion of these two images is the main process involved in stereopsis [5]. In addition, monocular cues, such as perspective, interposition, and texture gradients, can also aid in stereopsis [6]. Absolute disparity refers to the angles of one point’s projections on the left and right eyes with reference to each eye’s fovea, while the relative disparity refers to the difference of their absolute disparities between two eyes [7]. It has become clear that stereoacuity, the smallest detectable depth difference, mainly relies on the relative disparity [8, 9]. Thus, the term ‘binocular disparity’ in this paper refers to the relative disparity. Previous studies have investigated the correlation between neural and haemodynamic responses to stereoscopic stimuli [3] and also implemented fNIRS in an immersive virtual reality environment [10]. However, the associated visual fatigue has not been quantitatively studied. In fact, a low-quality artificial stereoscopic environment may cause various visual fatigue symptoms (e.g. headache, eye ache) and even irreversible health damage such as manifest esotropia [11]. A quantitative characterization of visual fatigue thus becomes increasingly important when in the pursuit of better quality for commercial 3D displays and virtual reality. Here, we apply fNIRS to measure the neural response to stereoscopic stimuli and further try to objectively evaluate the corresponding visual fatigue.

Experimentally, there are various methods to initiate and maintain the experience of stereovision, e.g., stereoscopic or auto-stereoscopic displays, dynamic or static random-dot stereograms (RDS), with or without the help of 3D glasses. The factors underlying visual fatigue during stereopsis depend on both the properties of the stimuli and how the participants interpret depth cues. Explicitly, the causes of visual fatigue during stereopsis mainly include anomalies of binocular visions, dichoptic errors, conflict between vergence eye movement and accommodation, and excessive binocular parallax [6]. In the present study, we make a comparison between different degrees of disparities, which should lead to different degrees of discomfort, to obtain an objective evaluation of visual fatigue and a more comfortable disparity range. The use of 3D glasses enables stereopsis, while the generation of binocular depth perception under natural viewing conditions is dependent on both voluntary eye movements and cognitive processing. The present study adopts a natural viewing method to reveal how the brain is engaged in the process of binocular depth perception and to examine the fatigue associated with it.

Researchers frequently use RDSs to study the neural correlates of stereopsis which can be triggered using binocular disparities. Dynamic RDSs can provide rich stereoscopic stimuli, but cannot completely replace monocular cues such as shade, motion, and occlusions [12]. In comparison, a static RDS provides depth cues that can be only obtained by binocular disparity, thus making the static RDS an optimal stimulus to study the effect of binocular disparity on visual fatigue. Research has found that several brain areas are involved in the processing of binocular depth perception and stereopsis. In one such study, single-unit recordings from macaque brains indicated that the primary visual cortical area (V1) and three extra striate cortical areas—the secondary visual area V2, the ventral extra striate area V4, and the dorsal extra striate area (V5, or middle temporal area)–featured cells that responded to disparity [5]. By measuring blood-oxygen-level-dependent signals with functional magnetic resonance imaging (fMRI) in the human brain, some studies further confirmed that stereoscopic depth perception in humans is a multi-stage process that involves both the dorsal and ventral cortical pathways, with each pathway playing a different role in perceptual processing [13]. Nevertheless, it is widely accepted that V1 and the parieto-occipital regions show more adaptations for depth perception [14–16]. On the other hand, it was reported that the dorsal pathway was more correlated with visual fatigue caused by excessive disparity [12] and showed exclusive function for generation of control signals for eye and hand movements [5]. Therefore, we focus on V1 and the dorsal parieto-occipital regions to study the depth perception process and its associated visual fatigue. It should be noted that the dorsal parieto-occipital portion is a common neural processing region for binocular depth perception when viewing either dynamic or static stereopsis [17, 18]. As a limitation, fNIRS can only collect cortical signals within 15~25mm beneath the scalp.

In the literature, Emoto et al. [19] and Li et al. [20] have used electroencephalography (EEG) with high-quality temporal resolution to measure the delay in transmission of visual information due to visual fatigue. Hagura et al. [21] have utilized a measurement tool that combines fMRI with magneto encephalography (MEG) to evaluate visual fatigue during 3D experiments. In the present study, to explore the possibility of evaluating visual fatigue by fNIRS, we assume that an HbO curve could be modeled by some kind of response function (a response function must be zero before stimulus onset, rise when viewing the RDS, and return to zero after stimulus offset; in this paper we use the normal distribution function as an approximation), and then we fit HbO curves to these response functions. Such an assumption is in agreement with typical fNIRS wave forms and also coincides with the purpose of first-order baseline corrections of HbO curves before computing Z scores [22]. In comparison, fitting parameters of HbO curves could provide not only amplitude and latency information similar to Z scores, but also areas, widths, and particularly multiple peaks which may reveal more processes of binocular depth perception. We note that visual fatigue is caused not just by stereopsis, but also by pure visual stimuli without any stereoscopic information. When the effect of binocular disparity on visual fatigue is considered, visual fatigue caused by pure visual stimuli is always present. The current study considers the total visual fatigue during stereopsis, leaving analysis of the disparity-induced contribution to future studies.

## Method

### Participants

A total of 13 participants (6 females and 7 males) were recruited who, upon viewing an RDS, were able to obtain stereopsis rapidly using binocular accommodation and vergence. All participants were right-handed and had normal or corrected-to-normal vision. The age of participants ranged from 21 to 29 (24.07 ±1.98). Participants avoided alcohol, caffeine, and cigarettes for 12 hours prior to the experiment. Before the experiment, participants were classified into two groups according to eye dominance [23]. To do this, participants were instructed to open both eyes and stare at a red dot on a display one meter away. Then, they positioned one finger in front of their eyes and tried to make the finger appear collinear with the red dot. After that, participants were instructed to close each of the eyes in turn. If the red dot remained in the same position, the open eye was dominant. In the present study, there were 2 left-eye-dominant and 11 right-eye-dominant participants. We have previously reported that cortical activation patterns for stereopsis were found to be different between left-eye-dominant and right-eye-dominant participants [24]. There was a left lateralization effect for right-eye-dominant participants. Therefore, only data from the 11 right-eye-dominant participants were included for further analysis.

During the experiment, participants were seated in a comfortable chair in front of a 19-inch computer screen with their chins placed on a chin rest so that the distance between their nasion and the center of the display would be fixed at 30cm. All participants gave written, informed consent.

### Experimental stimulus and fNIRS setup

**Fig 1:**
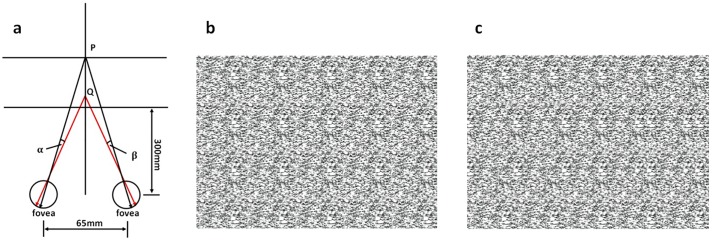
Binocular disparity and RDS. (a) Binocular fusion produces the sensation that a disc, Q, is floating in front of the background plane, P. The screen was 300mm away from nasion of participants. RDS with different disparities used in the experiment: for (b), the disparity was 1.5° and for (c) the disparity was 0.1°. Compared with (c), the depth of focus of the stereoscopic vision is lager for (b).

In this experiment, static RDS pairs were used. An RDS pair usually consists of two identical but horizontally-separated images that are meant to be fed into two eyes. One of the images bears a slight shift to the left relative to the background of the RDS, and the other bears the same amount of shift to the right. This symmetric displacement gives rise to binocular disparities α+β and triggers the depth perception processes [7, 25], as illustrated in Fig 1a. Our RDSs consisted of arrays (180mm × 130mm, 640px × 460px) of pixels of 32 different grayscales (0~255, equidistant distribution). In the center, disc shapes with radii of 30mm, or 107px, had been horizontally shifted by different amounts to produce different disparities (0.1°, 0.3°, 0.5°, 0.7°, 0.9°, 1.1°, 1.3°, and 1.5°; see Fig 1b and 1c for example). These stereograms were of the same size and had similar brightness and background colors (black, 0.05cd/m^2^).

**Fig 2:**
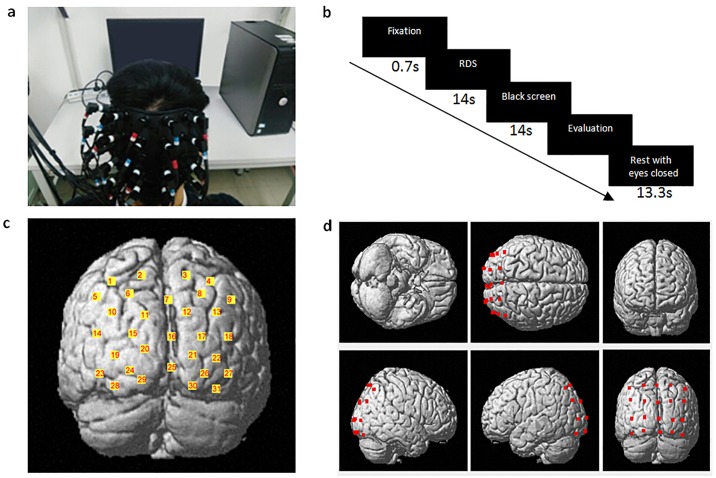
Experimental design and distribution of optodes. (a) The optodes covered on the scalp. (b) Task design following a slow event-related paradigm; each trial was composed of: a fixation (0.7s), RDS viewing and maintenance (14s), black screen (14s), subjective assessment (the duration depended on the participants’ response) and a shut-eye rest (13.3s). (c) The estimated cortical locations of 31 fNIRS channels. (d) The estimated cortical locations of the 20 optodes.

An fNIRS system (FOIRE-3000, Shimadzu, Kyoto, Japan) with three wavelengths (780nm, 805nm, and 830nm) of near-infrared light and with a sampling rate of 14.3 Hz (time resolution = 70 ms) was used to assess the changes in concentrations of HbO, Hb, and HbT in the occipital cortex and the parieto-occipital cortices of the brain. We fixed the optodes (optical sources and detectors) on the scalp using a helmet. As shown in Fig 2a and 2c, in order to ensure optode placement consistency across participants, we used the international 10–10 system [26]. We placed the column of midline optodes along the CZ-OZ line and located the lowest optode on the inion. The entire measured area was 9cm × 12cm, consisting of a 4 × 5 array with an inter-optode distance of 3cm. To further confirm the spatial location of the measurement channels (i.e., inter-optode paths from sources to detectors), a 3D digitizer (FASTRAK-Polhemus, USA) was used to record the exact spatial location of each optode and the anatomical landmarks on each participant’s head: nasion (Nz), central (Cz), and left and right pre-auricular points (LPA and RPA). According to the algorithms by Singh et al. [27], we obtained an estimated MNI (Montreal Neurological Institute) coordinate for each channel by converting the 3D spatial location data obtained from our 3D digitizer. The fNIRS channels in this study covered the dorsal parieto-occipital cortex and the primary visual cortex (V1) over Brodmann Area 17.

### Experimental protocol

During the experiment, participants were instructed to keep their heads still. At the start of the experiment, participants viewed 8 RDSs with 8 binocular disparities represented in a random order as a training session. After the training session, each RDS with a certain disparity was presented three times in a random order. There were thus 24 total trials. Each trial consisted of 4 parts, as shown in Fig 2b (0.7s staring fixation, 14s stereopsis viewing and maintenance, 14s black screen, subjective assessments, and 13.3s of rest with eyes closed). The rest period with eyes closed was designed for participants to recover between trials. An auto sound reminder provided by E-Prime (V2.0) was used at the beginning of the next trial. During the subjective assessments, four items (with 7 Likert scales) were to be evaluated: the stereopsis—perception of stereopsis, represented as the perception of the depth of focus between the foreground (disc) and the background (1: not stereoscopic; 7: very stereoscopic); the sustainability—effort required to sustain stereovision (1: easy to sustain, 7: hard to sustain); the clarity—clearance of the circular profile (1: poor clarity, 7: good clarity); and the discomfort—the degree of visual discomfort after RDS viewing (1: no discomfort, 7: much discomfort). Sustainability and discomfort were supposed to be the cause and result of visual fatigue and thus were subjective evaluations of visual fatigue. The impracticability of subjective evaluation of the ratio of disparity-induced contribution to visual fatigue is a major reason why the present study considers the total visual fatigue during stereopsis. The experimental protocol was approved by the Ethics Committee of South China Normal University.

### Data analysis

Since HbO signals were widely reported [28, 29] to outperform Hb signals in statistical analysis, only the HbO data was used for analysis in this study. First, we used the NIRS-SPM [30, 31] to analyze the data. This SPM-based software package for fNIRS data analysis was based on the general linear model (GLM) [32] and allowed us to estimate the neural activity for eight different disparities independently [33]. Its haemodynamic response function (HRF) filter and the wavelet-MDL (minimum description length) detrending algorithm were used to remove possible physiological interferences [20, 34, 35]. The beta values of the GLM for different trials were extracted as weights to account for the brain activity. Topography (based on the beta values) was plotted based on the location of the channels. The regions of interest (ROIs) were selected according to the most salient regions in topography.

**Fig 3:**
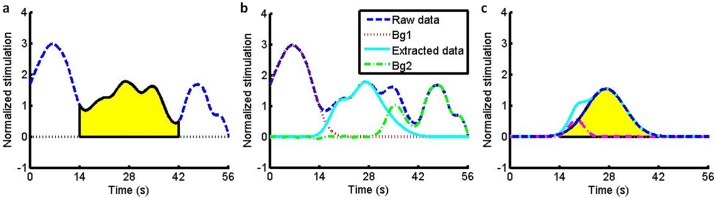
Illustration of the data processing. (a): a bad trial sample with large background noise; (b): extracted brain activity from the raw data; (c): fitting the data by two normal distribution functions.

To analyze the time course of the HbO data, we selected the time window of interest as the period of RDS viewing and black screen (28s for each trial) and reconstructed it using the following steps. Firstly, we normalized the HbO data by the standard deviation of each channel and subtracted the local minimum of each trial to obtain the time-course HbO curve, shown as a dashed blue line in Fig 3a, based upon which we could immediately calculate the total HbO intensity (S_0_) within the time window, shown as the yellow area. HbO intensity is referred to hereafter as the evoked response, and the time-course HbO wave form is defined as the response curve. Secondly, worth noting are that the background signals beyond the time window, caused either by insufficient rest between trials or some other stimulus-unrelated noise, significantly exaggerated the actual evoked response. To remove contamination from these background signals, one could either use the zero-order baseline correction to subtract a mean value of 2s pre-stimulus period or use the first-order baseline correction [22] to subtract a linear fitting of mean values of 2s pre-stimulus and 2s post-stimulus periods. However, here we preferred a novel baseline correction method: to fit the data before the time window into a smooth curve (Bg1), shown as the dotted red line in Fig 3b, and then subtract it from the original dashed blue curve. The remaining curve still bore background signals (Bg2) after the time window, and hence we fitted the data within the time window into another smooth (solid cyan) curve and extracted it as the effective response curve for further analyses. During these curve fittings, the normal distribution function N was used as an approximation of the response function, and four independent functions were adopted together to ensure the fidelity:

$$data=\sum_{i=1}^{4}S_{i}N(\mu_{i},\sigma_{i},t)$$ (1)

where *μ* was the position of a peak, *σ* was its width, *S* was its area, and *t* was the time variable. The effective response curve was zero just before stimulus onset (at 14s), increased when viewing RDSs, and decreased during the black screen period, and thus was much more in agreement with the hypothesis of the activation wave form, as shown in Fig 3c. Though the first-order baseline correction could remove those background signals and yield similar curves (zero prior to stimulus onset, rise when viewing, and return to zero after stimulus offset), we emphasize that, according to our assumption (HbO curve can be modeled by response functions), our baseline correction method is better because stimulations emerging in the black screen period (the first peak of Bg2 in Fig 3b) which are stimulus-unrelated and should not be taken into account, as we did, cannot be removed by the first-order baseline correction. Thirdly, one could visually recognize that there were two components of the wave form, including two peaks in the effective response curve. We then fitted them to two normal distribution functions:

$$HbO_{eff}=\sum_{i=1}^{2}S_{i}N(\mu_{i},\sigma_{i},t)$$ (2)

and obtained the area *S*, width *σ*, amplitude *α = SN(μ*, *σ*, *μ)* and position *μ* of each component of wave form respectively, as shown in Fig 3c. Parameters of position (latency) and amplitude also have applications in the Z-score method. Width (duration) *σ* indicates how long a process lasts, and area *S* indicates the accumulated physiological effect of a process which makes it a promising candidate to describe the visual fatigue.

To analyze the effect of disparities on subjective assessments, strength of activations (beta value), and properties of wave forms, we performed repeated measures analysis of variances (ANOVAs). The repeated measures ANOVA was conducted with eight levels (eight different disparity stimuli) of within-subject factors as independent variables. Subjective assessments, strength of activations (beta values) in each ROI, and properties of wave forms were used as dependent variables. Huynh-Feldt correction epsilon was used to correct the degrees of freedom in cases when Mauchly’s sphericity test for normality was violated. Subsequent post-hoc analysis with pairwise comparisons and paired-sample t-test were corrected by false discovery rate (FDR) [36]. We believe this is an optimal method to take the interaction of different variables into account, improving the efficiency of the statistics and preventing errors resulting from multiple comparisons.

We analyzed the relationship between subjective assessments and brain response (including activation strength and activation wave forms) at the group level. We firstly calculated the Pearson correlation coefficient *r* between subjective assessments, the strength of activation (beta values), and characters of activation wave forms at each disparity. The averaged *r* values were then calculated for all disparities. Through the entire analysis, the averaged *r* values and t-tests were made by converting *r* values to *z* values with Fisher’s *r*-*z* transformation. The averaged *z* values were converted back to *r* values by inverse transformations.

## Results

### Subjective assessments

**Fig 4:**
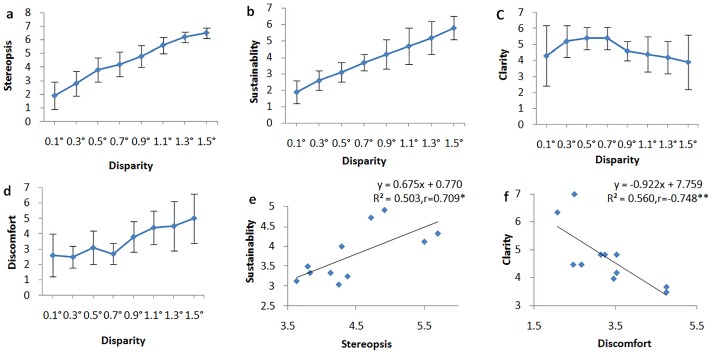
Statistical results of subjective assessments. Correlations between subjective assessments and different binocular disparities (a, b, c, d). The evaluation of sustainability is correlated with the perception of stereopsis (e) and the degree of discomfort (f). Subsequent post-hoc pairwise comparisons (with FDR controlled) reveal that the differences of evaluations are all significant or marginally significant (P_max_ = 0.052) among different disparities in (a), (b) and (d). The evaluation of RDS with 0.7° disparity is significantly larger than RDS with 1.1° in (c). *: Significance at the 0.05 level. **: Significance at the 0.01 level.

The results of ANOVA tests to determine the effect of disparities on the subjective assessments indicate that the degree of disparity has a significant influence on binocular depth perception (*F*_(7,10)_ = 97.898, *η*_*p*_^*2*^ = 0.907, *P* = 3.822 × 10^−14^), sustainability (*F*_*(7*,*10)*_ *= 62*.*751*, *η*_*p*_^*2*^ *= 0*.*863*, *P = 3*.*630 × 10*^*−16*^), clarity (*F*_*(7*,*10)*_ *= 5*.*556*, *η*_*p*_^*2*^ *= 0*.*357*, *P = 0*.*002*), and visual discomfort (*F*_*(7*,*10)*_ *= 8*.*381*, *η*_*p*_^*2*^ *= 0*.*456*, *P = 0*.*0002*). The subjective assessments of binocular depth perception and sustainability increase with the degree of disparity (Fig 4a and 4b). The evaluation of stereopsis is significantly correlated with sustainability (*r = 0*.*709*, *P = 0*.*015*, Fig 4e). In addition, the evaluation of clarity is significantly correlated with the degree of discomfort (*r = -0*.*748*, *P = 0*.*008*, Fig 4f).

### fNIRS data

**Fig 5:**
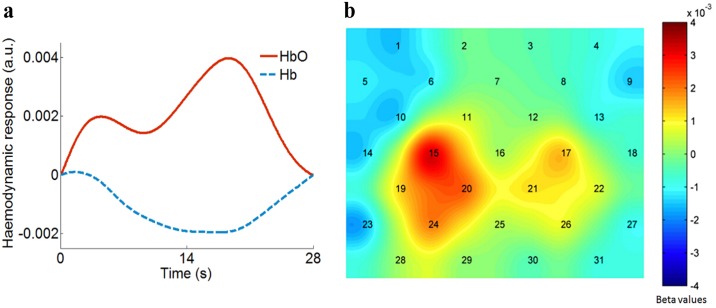
Haemodynamic response and beta values. (a): Comparison of HbO and Hb response to stereopsis. Averaged HbO signals (red solid curve) show larger magnitude and thus better sensitivity. (b): The topography of averaged beta values for 8 disparities from all participants (n = 11). The topography shows that the occipital cortex is spatially correlated with stereoscopic vision and that the activation pattern is associated with eye dominance.

For raw fNIRS data before NIRS-SPM processing, we averaged the time-course haemodynamic response over objects, trials, and channels. The noise level was derived as the standard deviation among 10 time points in a stationary plateau; it was 0.0004 with arbitrary units for both HbO and Hb signals. The mean value of signals within the time window was 0.0021 for HbO and -0.0011 for Hb. Dividing mean values by the standard deviation, we obtained a signal-to-noise ratio of 5.25 for HbO and 2.75 for Hb. After NIRS-SPM processing, the variation in HbO and Hb signals within the time window is shown in Fig 5a. We see that the Hb curve, with a smaller magnitude, evolved in the opposite direction to the HbO curve. The above characteristics of the HbO and Hb signals are consistent with many other findings in the literature [29, 37].

The topography of the averaged beta values for 8 disparities for all participants is shown in Fig 5b. Binocular depth perception leads to an increased HbO in the occipital cortex, especially in regions of V1 near PO3 (Ch15) and PO4 (Ch17). Compared with ROIs from the fMRI literature, regions of activation in the present study show an overlap in V1 and V2. Obviously, there is more activation in the left hemisphere than in the right hemisphere. According to the pattern of topography, we selected eight channels with the highest statistical activation, including four channels in the left hemisphere (#15, 19, 20, and 24) and four channels in the right hemisphere (#17, 21, 22, and 26), as two ROIs for further statistical analysis. The MNI coordinates of these channels are given in Table 1.

**Table 1:** The MNI coordinates of channels in two ROIs.

ROIs	Channel	Estimated MNI			Brain region	Probability
		X	Y	Z		
Left	15	-20	-103	18	V1 (BA17)	0.75
	19	-32	-100	5	V2 (BA18)	0.57
	20	-12	-108	7	V1 (BA17)	1
	24	-21	-107	-3	V1 (BA17)	0.90
Right	17	26	-101	16	V1 (BA17)	0.71
	22	36	-97	4	V2 (BA18)	0.80
	21	20	-106	6	V1 (BA17)	1
	26	27	-103	-5	V1 (BA17)	0.52

**Fig 6:**
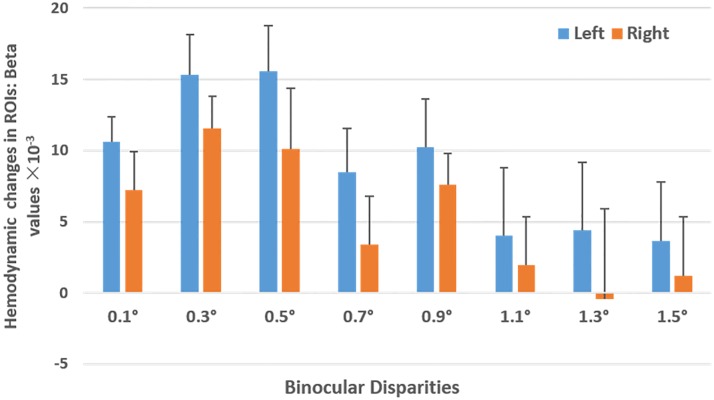
Haemodynamic changes in both ROIs. Haemodynamic changes in the left ROI (blue bar) and right ROI (orange bar) for RDSs with 8 disparities for all participants (n = 11). Error bars represent the standard error of the mean across all participants. Statistical analysis indicates that there is a left lateralization of the activation pattern; furthermore, haemodynamic response to an RDS with 0.5° disparity is significantly stronger than to an RDS with 1.1°.

Fig 6 shows the mean beta values for all participants (*n* = 11) for each ROI and the standard error of the mean across all participants. After performing a repeated-measures-ANOVA to test the effects of disparities and ROIs (left vs. right occipital cortex) on the haemodynamic changes of HbO (beta values), results indicate that there is a significant primary effect of ROI (*F*_*(1*,*10)*_ = 5.050, *P* = 0.048, *η*_*p*_^*2*^ = 0.336). Binocular depth perception leads to a significantly stronger activation in the left occipital cortex than in the right occipital cortex. The main effect of disparities is marginally significant (*F*_*(7*,*10)*_ = 2.245, *P* = 0.055, *η*_*p*_^*2*^ = 0.183). Subsequent post-hoc pairwise comparisons (with FDR controlled) reveal that the haemodynamic response in the occipital cortex to an RDS with 0.5° disparity is significantly stronger than that observed in response to an RDS with 1.1° disparity (*P* = 0.002). Haemodynamic response to an RDS with 0.3° disparity is marginally stronger than to an RDS with 1.1° (*P* = 0.008). The interaction effect between disparities and ROIs is not statistically significant (*F*_*(7*,*10)*_ = 0.470, *P* = 0.853, *η*_*p*_^*2*^ = 0.045).

Similarly, we performed an additional 6 repeated-measures-ANOVA tests to determine the effects of disparities and ROIs (left vs. right occipital cortex) on the properties of the time-course curve: Amplitude1, Area1, T1 (the time of the first peak), Width1, Amplitude2, Area2, T2 (the time of the second peak), and Width2. The results show that the interaction effect of ROIs and disparities on Area1 (*F*_*(7*,*10)*_ *= 3*.*777*, *η*_*p*_^*2*^ *= 0*.*274*, *P = 0*.*011*), Amplitude 1 (*F*_*(7*,*10)*_ *= 3*.*604*, *η*_*p*_^*2*^ *= 0*.*265*, *P = 0*.*003*), T2 (*F*_*(7*,*10)*_ *= 3*.*031*, *η*_*p*_^*2*^ *= 0*.*233*, *P = 0*.*037*), and Width2 (*F*_*(7*,*10)*_ *= 2*.*753*, *η*_*p*_^*2*^ *= 0*.*216*, *P = 0*.*043*), are significant. Subsequent post-hoc pairwise comparisons (corrected by FDR) indicate that, in the left ROI, Area1 (*P* = 0.002) and Amplitude1 (*P* = 0.003) subjected to a disparity of 1.3° are significantly and marginally significantly larger, respectively, as compared with the disparity of 1.5°. T2 of the disparity of 0.1° (*P* = 0.003), 0.5° (*P* = 0.001), 1.1° (*P* = 0.008), and 1.3° (*P* = 0.006) are significantly larger compared with the disparity of 0.9°, while Width2 of the disparity of 0.3° (*P* = 0.002), 0.7° (*P* = 0.008) are significantly and marginally significantly larger, respectively, as compared with the disparity of 1.5°.

Fitting parameters of the HbO curves are given in Table 2. We find that the position of the first peak (T1, around 6s) is consistent with the typical reaching-peak position (5-7s) in Ref. [10], and the position of the second peak (T2, >14s) is qualitatively consistent with the findings of Ref. [3]: that there is a period of high activation after stimulus offset. All parameters will be analyzed further in the following subsection.

**Table 2:** The mean and standard deviation of haemodynamic change in different ROIs (mean ± standard deviation).

	ROIs	
	Left	Right
Amplitude 1	1.210 ± 0.317	1.128 ± 0.308
Area 1	10.190 ± 2.186	9.244 ± 2.547
T1	6.252 ± 0.588	6.131 ± 0.899
Width1	3.345 ± 0.267	3.126 ± 0.178
Amplitude 2	1.918 ± 0.438	1.882 ± 0.507
Area 2	21.228 ± 5.479	20.522 ± 6.478
T 2	14.655 ± 0.732	14.760 ± 0.880
Width2	4.361 ± 0.430	4.234 ± 0.493
S_0_	50.186 ± 3.661	47.251 ± 3.859

### Correlation between subjective assessments and fNIRS data

Pearson correlation r analyses at the group level are shown in Table 3. Results show that the strength of activation (beta value) is not significantly correlated with subjective assessments in neither the left ROI nor the right ROI. In comparison, Area1 in the left ROI is significantly correlated with the assessment of discomfort during binocular depth perception; Amplitude2 is significantly correlated with subjective experience of stereopsis in both the left and right ROI. Widths show no significant correlation with subjective assessment and thus are not given in the table.

**Table 3:** The correlation between subjective assessments and fNIRS data at group level (n = 11).

ROIs		Pearson Correlation Coefficient r			
		Stereopsis	Clarity	Sustainability	Discomfort
Left	Beta	0.05	-0.04	-0.09	0.31
	S0	0.18	-0.10	0.03	0.26
	Amplitude 1	-0.27	-0.10	0.39	0.53
	T1	0.18	-0.38	0.16	0.15
	Area 1	-0.18	-0.25	-0.13	0.56*
	Amplitude 2	0.58*	-0.12	0.38	0.36
	T2	0.23	0.10	0.23	0.01
	Area 2	0.34	0.07	0.07	0.14
Right	Beta	-0.24	-0.12	-0.14	-0.22
	S0	0.22	0.05	-0.04	0.05
	Amplitude 1	-0.08	0.07	0.04	0.11
	T1	0.18	-0.13	0.16	0.15
	Area 1	-0.04	0.04	-0.01	0.03
	Amplitude 2	0.59*	0.36	0.27	-0.01
	T2	0.05	-0.10	0.11	0.03
	Area 2	0.52	0.06	0.45	-0.01

* Significance at the 0.05 level.

Based on the above results, we interpret that the first peak reflects the process of generating the stereopsis, and the second peak reflects the process of sustaining stereoscopic perception. Since discomfort is significantly correlated with the left Area1, we can conclude that visual fatigue is mainly caused by generating stereovision, but not by sustaining it. Once the stereovision is established, haemodynamic response approaches a peak with an amplitude correlated with and determined by stereopsis. Though on average the response becomes larger (Amplitude2 > Amplitude1, see Table 2), less discomfort is felt.

## Discussion

**Fig 7:**
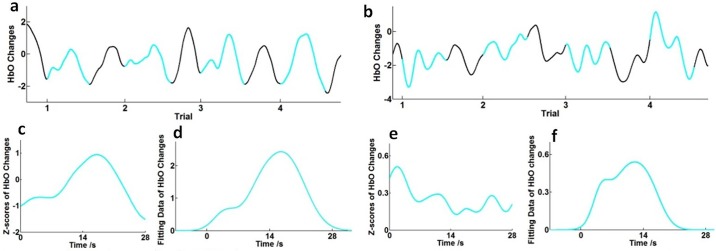
Comparison between different baseline correction methods. Good (a) and bad (b) trial samples after preprocessing of fNIRS data. Solid cyan curves: the time window for RDSs viewing and black screen (28s for each trial). Black curves: subjective assessments and shut-eye rests (13.3s). The averaged response of good (c) and bad (e) trial samples using traditional time-course analysis (zero order baseline corrections). The averaged response of good (d) and bad (f) trial samples using curvilinear fitting baseline corrections.

One of the major issues to be considered when analyzing the event-related fNIRS data is how to account for artifacts and filter them out. As shown in Fig 7, we extracted two time serial data containing 4 trials for each series. After using the HRF filter and the wavelet-MDL detrending algorithm to remove physical noise and artifacts for each time series, most of the HbO-evoked curves of stimuli during the time window (28s for each trial) include two peaks (similar to Fig 7a) and baseline drifts. As mentioned in the Method section, we believe our curvilinear (response function) fitting baseline correction method is better than zero-order or first-order (linear fitting) baseline correction. Here we shall enhance our argument with further proofs. Because of insufficient rest or other stimulus-unrelated factors, there are trials, like Fig 7b, where HbO curves are obviously tampered by trends of background signals beyond the time window, though the time for rest is sufficiently long. Under our assumption, HbO curves should be a superposition of response functions, and thus we believe HbO signals after stimulus onset are the superposition of actual haemodynamic responses to RDS and the tails of the background waves. This could especially explain negative trends of dropping waves (e.g. trials 1 in Fig 7b): at the beginning of stimuli, the actual haemodynamic response to RDS is rising but too small compared with the background signals, and thus the total HbO curve drops as the tail of the background wave. We emphasize that the negative trends of HbO could not be treated as negative responses to RDS since the curve would drop even if the stimulus is not turned on at the required time. As a comparison with traditional time-course analysis where zero-order baselines were subtracted in each trial and then the data is converted to Z scores, averaged results for 4 trials are given in Fig 7c–7f. We see that our curvilinear fitting baseline corrections show trivial differences with traditional baseline corrections for good trials (c, d), but considerably improve the performance for bad trials with large background artifacts (e, f).

In our attempt to model an effective HbO curve within the time window by two response functions, we have demonstrated the ability of this method to analyze two or more processes together, which we believe is important and might benefit future research. With this fitting method, we have recovered the first-peak latency consistent with previous paper in the literature [10]. Total evoked response S_0_ and maximum Z-score Amplitude2, which could be derived by the traditional method, show no significant correlation with visual fatigue. Our fitting parameter Area1, which cannot be obtained by the Z-score method, is significantly correlated with discomfort and could be used to quantify visual fatigue. That being said, the heuristic fitting method still needs more investigation to test its validity. Furthermore, the normal distribution (Gaussian distribution) function, due to its symmetry and never attaining a zero value, may not be the ideal choice for the response function. Other types of response functions could be studied in the future.

Results of subjective assessments (Fig 4) show that participants are sensitive to disparities. As the binocular disparity increased, participants reported greater perception of stereopsis and clarity, as well as increased difficulty and discomfort in sustaining the stereopsis. However, the fNIRS data does not show a similar sensitivity across the disparities. A possible interpretation is that the regions of our measurement are not directly related to 3D shapes (the intraparietal sulcus) [38–40] and eye movement (the frontal eye field and intraparietal sulcus) [41]. Also, for the limited cortical depth and channels allowed by NIRS, we could only assess the haemodynamic response of the cortex and are unable to examine the roles of other important brain regions, such as the intra-parietal sulcus. Thus, further studies could examine the role of the eye movement area of the brain during stereopsis. Nevertheless, our results support the hypothesis that the primary visual cortex plays a partial role in eye movement and the processing of depth cues. Furthermore, converging the results in Figs 4c, 4d and 6, compared with the disparity beyond 1°, brain activation for disparities within 1° leads to more brain activation, better clarity, and less visual discomfort, in agreement with previous studies [6, 42, 43]. A possible reason for this might be that an RDS over 1.1° would cause too much visual fatigue and overload to brain area correlated with stereoscopic vision. We suppose that a disparity around 0.5° is optimal for stereoscopic vision, which leads to the highest cortical response and produces little discomfort.

## Conclusion

In summary, by using fNIRS to measure the haemodynamic response during stereopsis, we have determined that the occipital cortex is spatially correlated with binocular depth perception. By using the fitting method, we have successfully distinguished two steps in the process of depth perception and found that visual fatigue is mainly caused by generating, but not sustaining, stereovision. Haemodynamic response during sustaining stereovision has been found to be correlated with stereopsis. Combining statistical parameter analysis and the fitted time-course analysis, fNIRS could be a promising method to study visual fatigue and possibly other multi-process neural bases.
